# Surgical treatment of sacroiliac joint infection

**DOI:** 10.1007/s10195-013-0233-3

**Published:** 2013-04-05

**Authors:** Hamdan Ahmed, Ahmed Ezzat Siam, Gouda-Mohamed Gouda-Mohamed, Heinrich Boehm

**Affiliations:** Department of Spinal Surgery and Paraplegiology, Zentralklinik Bad Berka, Robert Koch Allee 9, 99438 Bad Berka, Germany

**Keywords:** Sacroiliac joint infection, Pyogenic sacroiliitis, Tuberculous sacroiliitis, Sacroiliac fusion

## Abstract

**Background:**

Sacroiliac joint infection is rare and frequently missed; purpose of this study is to describe the clinical presentations, comorbidities, laboratory and imaging findings, surgical options and outcomes of this rare condition.

**Materials and methods:**

We reviewed all cases of surgical treatment of sacroiliac joint infection operated at our institution between January 1994 and December 2011. Twenty-two patients were included: 14 females and 8 males, with mean age of 50 years. The mean follow-up period was 34 months. Twenty-four operations were performed. Coinciding infection was found in 11 cases (50 %). Twelve patients (54.5 %) presented acutely, while ten patients (45.5 %) had chronic infection.

**Results:**

Tuberculous infection was diagnosed in 5 cases and nonspecific infection in 13 cases. In four cases, no organism was isolated. Eleven cases were subjected to debridement only, while debridement and arthrodesis was needed in 11 cases. Eight patients had excellent clinical results, five good, three fair and four poor; one patient was lost to follow-up, and one patient died after 2 weeks. The operative technique depended on the course of the infection, bone destruction and general condition of the patient. There was a significant change in C-reactive protein and erythrocyte sedimentation rate preoperatively and 6 weeks postoperatively, while the difference in white blood cell count was nonsignificant.

**Conclusions:**

In acute cases, the primary aim should be to save joint integrity by early debridement, depending on joint destruction and general patient condition. When it is chronic, it is not secure only to debride the joint, which should be fused.

## Introduction

Isolated sacroiliac joint (SIJ) infection is rare. Between 1878 and 1990, only 166 cases were documented in the English-language literature [1], although pyogenic sacroiliitis is estimated to account for 1–2 % of cases of septic arthritis or bone infection [2]. Skeletal tuberculosis accounts for 3–5 % of all tuberculosis, of which approximately 10 % occurs at the SIJ [3]. Predisposing factors include intravenous drug abuse, immune suppression, pregnancy, trauma and infection elsewhere in the body [4]. However, in over 40 % of patients, the primary site of infection may never be identified [1, 5]. Clinical findings may be obscured, but usually include buttock pain and limping. In severe cases, the patient may be unable to find a comfortable position in bed and demonstrates a positive flexion, abduction and external rotation (FABER) test of the hip joint that dramatically aggravates the pain. Fever is not a constant finding [6]. Accurate diagnosis is frequently delayed due to lack of awareness of the condition by clinicians, non-specific clinical presentation and poorly localising signs of infection; mimicking features of septic arthritis of the hip, osteitis of the ilium and lumbar disc herniation [7–9]. Magnetic resonance imaging (MRI) has been proved to be the best tool for early diagnosis of SIJ infection. MRI findings in the acute phase are intra-articular fluid, subchondral bone marrow oedema, articular and periarticular post-gadolinium enhancement and soft tissue oedema, and in the chronic phase: periarticular bone marrow reconversion, replacement of articular cartilage by pannus, bone erosion, subchondral sclerosis, joint space widening or narrowing and ankylosis [10]. The purpose of this study is to describe the authors’ experience regarding the clinical presentations, comorbidities, laboratory and radiological findings as well as operative options and postoperative outcome of sacroiliac joint infections.

## Materials and methods

This is a retrospective clinical study in a single facility. Between January 1994 and December 2011, 22 patients were operated in our institution for treatment of sacroiliac joint infection. Cases of non-infectious sacroiliitis and conservatively treated infections were excluded from this study.

The criteria for diagnosis were: clinical; local pain and tenderness in the SIJ, limping, clinical manifestations and laboratory findings suggesting infection [chemical: elevated white blood cell (WBC) count, C-reactive protein (CRP) and/or erythrocyte sedimentation rate (ESR); and microbiological: positive blood and/or intraoperative culture], in association with early MRI and late radiographic changes in the SIJ (periarticular bone destruction and cavitation, joint space widening, sclerosing); all confirming the diagnosis. Cases of non-specific infection were considered acute when presenting within 1 month of onset of clinical symptoms and chronic when presented later. All tuberculous cases were chronic.

The mean follow-up (FU) period was 34 months (6–90 months). One patient was lost to FU, and one patient died 2 weeks after surgery due to multiple organ failure.

Clinical examination, laboratory investigations and plain radiographs were done routinely: preoperatively, 1 day and 2 weeks postoperatively and at the FU visits (6 weeks, 3 months, 1 year postoperatively and then every 2 years). Patients were followed up by their family physicians for clinical or laboratory changes. MRI was done preoperatively, after 3 months and 1 year (and when recurrence was suspected). Computed tomography (CT) was needed preoperatively only in nine cases for assessment of bone destruction and postoperatively for assessment of bony fusion, only when symptomatic.

Surgery was indicated (from senior author’s experience, H.B.) in cases of failure of conservative measures, abscess formation from the beginning, bone destruction, septicaemia or neurological deficits.

All patients underwent operative treatment in the form of debridement with or without joint arthrodesis. The surgical approach was either posterior, anterior or combined anterior and posterior. The localisation of the infection (abscess and soft tissue infiltration) as demonstrated by MRI dictated the operative approach.

Postoperative treatment included culture-based antimicrobial therapy or broad-spectrum antibiotic therapy when no organism was isolated, for 6 weeks in non-specific infections and 6–12 months in tuberculous infections.

We concluded the final functional outcome by questionnaires including Odom’s criteria [11] that categorised patients’ satisfaction into four grades of excellent, good, fair and poor as follows:

- Excellent: all preoperative symptoms relieved, abnormal findings unchanged or improved;
- Good: minimum residual of preoperative symptoms not requiring medication or limiting activity, and abnormal findings unchanged or improved;
- Fair: definite relief of some preoperative symptoms with others remaining unchanged or only slightly improved;
- Poor: symptoms and signs unchanged from preoperative status or worse.

The infection was considered to be healed by the disappearance of clinical symptoms (pain, fever, fistula etc.) and laboratory parameters of infection (WBC, CRP and ESR) as well as radiographic and MRI confirmation of subsidence of infection (disappearance of bone oedema, abscess resolution etc.).

The joint was considered to be fused by the following radiographic criteria (when fusion is doubtful, follow-up CT after 1 year is advisable):

1. Absence of radiolucency crossing the entire joint space
2. Side-wall fusion and inter-run fusion
3. Absence of loosening or metal compromise in plain radiographs
4. Clinically: absence of local symptoms of the joint (pain and tenderness)

Descriptive statistics were determined by calculation of the mean, standard deviation and range. Statistical analysis was needed to compare the preoperative laboratory findings versus the 6-week postoperative values using the Wilcoxon signed-rank test, and statistical significance was defined as *p* < 0.05.

This study has been approved by the institutional ethics committee in accordance with the ethical standards laid down in the 1964 Declaration of Helsinki. All persons included in the study gave their informed consent to have their data and diagnostic findings involved in medical research prior to their inclusion in the study.

## Results

Twelve patients (54.5 %) presented acutely, while ten patients (45.5 %) had chronic infection (Table 1). Marked weight loss was reported by two patients (9.1 %). At time of admission, coinciding infection was found in 11 cases (50 %), of which 6 cases were spondylodiscitis and 1 case was epidural abscess. Eight patients had received antimicrobial therapy.

**Table 1:** Demography, associated infections and comorbidities

Case	Age (years)	Sex	Main presentation	Other infections	Comorbidities	Previous operations	Affected side	Course
1	42.5	M	Fistula	Pulmonary tuberculosis, epididymitis	None	None	Left	Chronic
2	42.4	F	Fistula	Spondylodiscitis L5–S1	None	Multiple curettage operations before 6 months	Left	Chronic
3	63.1	M	Acute paraplegia	Spondylodiscitis T7–8, acute necrotising cholecystitis	Incomplete paraplegia sub T7, diabetes mellitus	T7–8 fusion before 2 months	Left	Acute
4	56	M	Fistula	Psoas abscess^a^	None	Multiple operations in SIJ	Right	Chronic
5	24.8	F	Local pain	Broncho-pneumonia, psoas abscess, staphylococcal septicaemia	Anorexia nervosa (body weight 36 kg)	None	Left	Chronic
6	68.8	F	Local pain	Spondylodiscitis L2–3, epidural abscess	Cardio-respiratory insufficiency, diabetes mellitus, morbid obesity	None	Right	Acute
7	64.1	M	Local pain	Psoas abscess^a^	None	None	Left	Chronic
8	44.1	M	Local pain	None	None	None	Right	Acute
9	30.3	F	Local pain	Staphylococcal septicaemia	None	None	Right	Chronic
10	63.3	F	Sciatic pain	None	Rectal carcinoma (radio- and chemotherapy)	Cortisone local injection	Right	Acute
11	61.4	F	Back pain	None	None	Seven operations in SIJ before 30 years	Right	Chronic
12	25.2	M	Difficult weight bearing	None	None	None	Left	Acute
13	65.6	F	Difficult weight bearing	Psoas abscess, epidural abscess	None	None	Left	Acute
14	45.9	F	Difficult weight bearing	None	None	None	Right	Acute
15	43.1	F	Acute paraplegia	Chronic leg ulcerations, incomplete paraplegia sub T9 with spondylodiscitis T9–10	None	None	Left	Acute
16	42	F	Local pain	None	Morbid obesity	Local injection	Right	Acute
17	79.6	F	Acute paraplegia	Spondylodiscitis L2–3	Morbid obesity	Bone graft before 2 years, same side	Right	Acute
18	68.5	F	Back pain	Candida sepsis, staphylococcal sepsis, sacral decubitus, acute bronchitis	Cardio-respiratory insufficiency, multiple organ failure, corticosteroid therapy	None	Left	Acute
19	44.3	F	Local pain	None	None	None	Left	Chronic
20	52.8	M	Local pain	Spondylodiscitis L5–S1, psoas abscess, sacral decubitus ulcer	Complete paraplegia sub T7, diabetes mellitus, morbid obesity	Myocutaneous flap before 7 years because of sacral decubitus ulcer	Bilateral	Chronic
21	16.6	M	Local pain	None	None	None	Left	Acute
22	54.7	M	Sciatic pain	None	None	None	Right	Chronic

^a^Psoas abscess alone was not considered as an associated infection because it is a part of the SIJ infection process itself

Radiographs were done preoperatively in all patients. In the acute stage of non-specific infections it appeared to be normal, while in chronic cases it showed blurring of the outlines of the sacroiliac joint, widening of the joint space, periarticular osteopaenia, sclerosis and erosion of the joint margins. MRI was done preoperatively for all patients. It demonstrated abscess formation in the piriformis, iliacus, gluteus or iliopsoas muscle as well as inflammatory signal changes in the surrounding soft tissues. Anterior capsule may be stretched or damaged. Other findings included: bone oedema, soft tissue infiltration and myositis. CT was done preoperatively in nine cases with chronic infection and showed joint space widening, sclerosis of the margins of the joint, cavitations and sequestrum formation (Tables 2, 3).

**Table 2:** Preoperative imaging and laboratory findings preoperatively and 6 weeks postoperatively in patients with non-specific infection

Case	Preoperative imaging			Preoperative lab			6 weeks postoperative		
	Radiographs	MRI	CT	WBC (/mm^3^)	ESR (mm/h)	CRP (mg/dL)	WBC (/mm^3^)	ESR (mm/h)	CRP (mg/dL)
3	Periarticular osteopaenia	Bone and iliacus and gluteal muscle oedema and abscess formation	–	13,700	70	87	8,400	12	21
5	Sclerosis and narrowing of joint space	Localised area of fluid in the joint	Sclerosis and cavitation	13,400	92	250	9,600	33	46
6	Normal	Abscess and oedema in gluteal muscle	–	10,600	89	117	7,100	19	57.2
7	Partially fused joint and localised area of cavitation	Localised cavity with fluid signal	–	7,800	79	27.5	9,000	83	18.7
8	Normal	Periarticular bone oedema, fluid signal in the joint and soft tissue	–	4,300	66	65.2	6,300	32	11.4
9	Narrow joint	Abscess formation and soft tissue and bone oedema	Joint narrowing and destruction	9,800	73	81.3	5,700	26	7.9
10	Sclerosis and cavitation	Posterior abscess formation	–	15,500	133	251.7	7,600	93	10.1
12	Normal	Periarticular oedema and fluid signal	–	19,700	64	255.4	8,700	55	13.3
13	Normal	Fluid signal in joint and bone oedema	–	10,900	83	90.5	6,900	64	16.9
14	Widening of the joint space	Fluid signal in the joint and periarticular oedema	Joint widening and sclerosis of the edges	12,200	128	135.1	7,900	29	12.6
15	Widening and cavitation of the joint surfaces	Abscess formation and bone and soft tissue oedema	Widening and localised cavitation	8,800	78	110	8,300	32	5.6
16	Wide joint with sclerosis	Abscess formation and soft tissue oedema	–	3,600	103	104	6,800	61	12.7
17	Wide joint	Tissue and joint fluid signal	Joint widening	11,800	74	153.2	7,600	71	55.6
18	Normal	Fluid in the joint and adjacent tissue anteriorly	–	13,600	86	79	27,400	51	65.3
19	Periarticular osteopaenia	Bone oedema and fluid signal in the joint	–	4,800	46	9.7	4,900	20	1.5
21	Normal	Fluid signal, periarticular and in the joint	–	10,100	77	258.7	7,300	39	16.8
22	Widening and cavitation	Abscess and soft tissue oedema posterior and anterior	Sclerosis and cavitation of the joint	5,000	38	7.6	5,900	73	13.1

**Table 3:** Preoperative imaging and laboratory findings preoperatively and 6 weeks postoperatively in patients with tuberculous infection

Case	Preoperative imaging			Preoperative lab			6 weeks postoperative		
	Radiographs	MRI	CT	WBC (/mm^3^)	ESR (mm/h)	CRP (mg/dL)	WBC (/mm^3^)	ESR (mm/h)	CRP (mg/dL)
1	Joint destruction and sclerosis	Fluid signal	–	5,100	59	38	7,300	81	30
2	Bone sclerosis and partially fused joint	Localised fluid signal in the joint	Fused joint with localised cavitation	4,600	95	48	5,200	32	13
4	Partially fused	Localised fluid cavity	–	5,600	112	40	7,100	42	15
11	Fused joint	Abscess above the joint	Fused joint with cavity	13,600	34	35.6	13,400	38	7.8
20	Partially fused joint	Fluid signal in the sacrum and parts of the joint	Sclerosis and cavitation of the sacrum	8,300	60	125.6	5,000	48	48.5

### Laboratory findings

In tuberculous infection, mean values were as follows: C-reactive protein (CRP) of 57.44 ± 38.39 mg/dL, erythrocyte sedimentation rate (ESR) of 72 ± 31.17 mm/h and white blood cell (WBC) count of 7,440 ± 3,729/mm^3^. Postoperatively, the mean CRP was 22.86 ± 16.54 mg/dL, ESR was 48.2 ± 19.24 mm/h and WBC was 7,600 ± 3,410/mm^3^ (Table 3). In non-specific infection, the mean CRP was 122.52 ± 84.74 mg/dL, ESR was 81.12 ± 24.32 mm/h and WBC was 10,329.4 ± 4,343/mm^3^, while postoperatively CRP was 22.69 ± 19.92 mg/dL, ESR was 46.65 ± 24.29 mm/h and WBC was 8,552.9 ± 5,012/mm^3^ (Table 2). The change was statistically significant for CRP and ESR (*p* < 0.001 and = 0.001, respectively), while in WBC the difference was nonsignificant (*p* = 0.082).

### Operative treatment

**Fig. 1:**
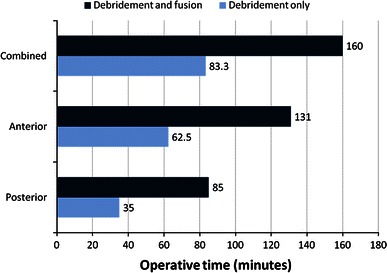
Diagram comparing the mean operative time of surgery

Eleven cases (50 %) were subjected to debridement only, while debridement and arthrodesis was needed in the other 11 cases. Two patients required revision because of recurrent infection (after complete healing); one was posteriorly debrided for the second time, and one had attempted fusion through anterior approach and was reoperated with a stand-alone cage; i.e. this study included 24 surgeries in the 22 reviewed patients (Table 4). The mean operative time for debridement without fusion was 35 min for posterior approach, 62.5 min for anterior approach and 83.33 min for combined anterior and posterior approaches, while in debridement and fusion it was 85, 131 and 160 min, respectively (Fig. 1).

**Table 4:** Operative and postoperative results

Case	Operation type	Approach	Fusion method	Operative time (min)	Blood loss (ml)	Causative organism	Antimicrobial therapy (months)	Follow-up (months)	Clinical outcome
1	Debridement and fusion	Posterior	Bone graft and screws	90	450	*M. tuberculosis*	Rifampicin + isoniazid (6)	25	Good
2	Debridement and sequestrectomy	Posterior	None	50	300	*M. tuberculosis*	Rifampicin + isoniazid (12^a^)	Lost	–
3	Debridement and fusion	Posterior and anterior	Bone graft	110	500	*S. aureus*	Clindamycin (3)	18	Good
4	Debridement	Posterior/posterior^b^	None	45/35	320/480	*M. tuberculosis*	Rifampicin + isoniazid (6)	6	Poor
5	Debridement and fusion	Posterior	Bone graft and screws	70	375	*S. aureus*	Ampicillin–sulbactam (2)	7	Excellent
6	Debridement	Posterior	None	30	175	*S. aureus*	Flucloxacillin (2)	8	Poor
7	Debridement	Posterior and anterior	None	60	1,000	No organism	Ciprofloxacin (3)	37	Excellent
8	Debridement and fusion	Posterior and anterior	Bone graft with cage and screws	130	600	*S. aureus*	Clindamycin (3)	90	Excellent
9	Debridement and fusion	Posterior and anterior	Bone graft with cage and screws	215	750	*S. aureus*	Clindamycin (3)	49	Excellent
10	Debridement	Posterior	None	60	190	*S. aureus*	Cefuroxime (3)	21	Good
11	Debridement	Posterior	None	15	70	*M. tuberculosis*	Ciprofloxacin (4), ethambutol + rifampicin + isoniazid + pyrazinamide (6)	86	Excellent
12	Debridement	Posterior and anterior	None	60	100	*S. aureus*	Flucloxacillin (3)	80	Excellent
13	Debridement	Anterior	None	45	300	*S. aureus*	Ciprofloxacillin (3)	61	Fair
14	Debridement and fusion	Anterior	Bone graft with cage	95	400	*S. aureus*	Clindamycin (3)	53	Fair
15	Debridement and fusion	Anterior/anterior^b^	Bone graft then bone graft with cage	270/160	300/700	*E. faecalis*	Flucloxacillin (3)	42	Poor
16	Debridement and fusion	Anterior	Bone graft with cage	100	100	*S. aureus*	Clindamycin (3)	15	Excellent
17	Debridement and fusion	Anterior	Bone graft	30	200	No organism	Ciprofloxacin (3)	8	Excellent
18	Debridement	Anterior	None	80	250	*S. aureus*	Clindamycin (2^a^)	Died	–
19	Debridement	Posterior	None	10	50	No organism	Flucloxacillin (2.5)	36	Fair
20	Debridement and fusion	Posterior	Bone graft	95	300	*M. tuberculosis*	Nitrofurantoin + rifampicin + isoniazid (6)	9	Poor
21	Debridement	Posterior and anterior	None	130	500	*S. aureus*	Flucloxacillin (0.5)	21	Good
22	Debridement and fusion	Posterior and anterior	Bone graft with screws	185	200	No organism	Clindamycin (3)	7	Good

^a^Both values indicate the intended period of antimicrobial therapy, which was interrupted by patient death or loss of FU

^b^Patients who underwent two operations

The causative organism was *Mycobacterium tuberculosis* in 5 cases (22.7 %), *Staphylococcus aureus* in 12 cases (54.5 %) and *Enterococcus faecalis* in 1 case. In four cases, no organism was isolated (Table 4).

The postoperative immobilisation period depended on the general condition of the patient and the operative technique. Postoperative treatment included culture-based antimicrobial therapy or broad-spectrum antibiotic therapy when no organism was isolated (Table 4).

### Outcome

Functionally, eight patients had excellent results (40 %), five good (25 %), three fair (15 %) and four poor (20 %) (Table 4).

Sound fusion was achieved in ten cases (50 %) within the first year after surgery; in the other ten cases, no signs of fusion were found in final radiographs.

Complications included recurrence of infection in two cases, delayed wound healing in three cases and chronic pain in three cases.

## Discussion

SIJ infection is a rare condition [1] which is usually associated with multiple predisposing factors and infection elsewhere in the body [4]. Clinically, it may be obscured by hip pain and poorly localising signs of infection with or without fever [6–9].

Despite the limitations of this retrospective study, including a relatively heterogeneous group of patients with a wide variation of preoperative conditions and surgical methods and the lack of similar studies to compare with, it represents the largest series of surgical treatment of this rare condition. It identifies the clinical, laboratory and radiological findings as well as surgical options and outcomes of this joint infection.

Bacterial infection of the SIJ is thought to occur most commonly by haematogenous spread [5, 12]. Vyskocil et al. [1] reviewed 166 reported cases of septic sacroiliitis and demonstrated that no associated factors were noted in 41 % of patients. In this series, there was an associated infection in 11 patients (50 %). Comorbidities were present in eight patients (36.36 %). The diagnosis of SIJ infection should be suspected in the presence of certain clinical, laboratory and radiological findings. The clinical symptoms are local sacroiliac pain, low back pain with or without sciatic pain, associated with inability to bear weight in most cases. On the other hand, fever was not a constant presenting symptom [6]. In our study, only four patients (18.2 %) had fever. Other presenting symptoms included fistula and abscess formation. On local examination, there was always tenderness on direct pressure over the joint with positive Gaenslen’s and FABER tests in all patients, which is consistent with the findings of Delbarre et al. [6] and Ramlakan and Govender [13].

Murphy et al. [14] showed that MRI in comparison with CT is both more sensitive for early diagnosis and superior in evaluation of cartilage integrity and early detection of osseous erosions in patients with inflammatory and infectious sacroiliitis. In our series, MRI was done in all patients preoperatively, while CT was done in only nine cases (40.1 %), in chronic cases for assessment of the extent of bony destruction and operative planning. Isotope bone scanning is a helpful tool for diagnosis; however, it has three main disadvantages: the inability to differentiate infectious from non-infectious sacroiliitis [2, 8, 12, 15], the inability to differentiate sacroiliitis from psoas or gluteal abscess and the inability to identify spread of the infection from the joint into the surrounding tissues [16].

**Fig. 2:**
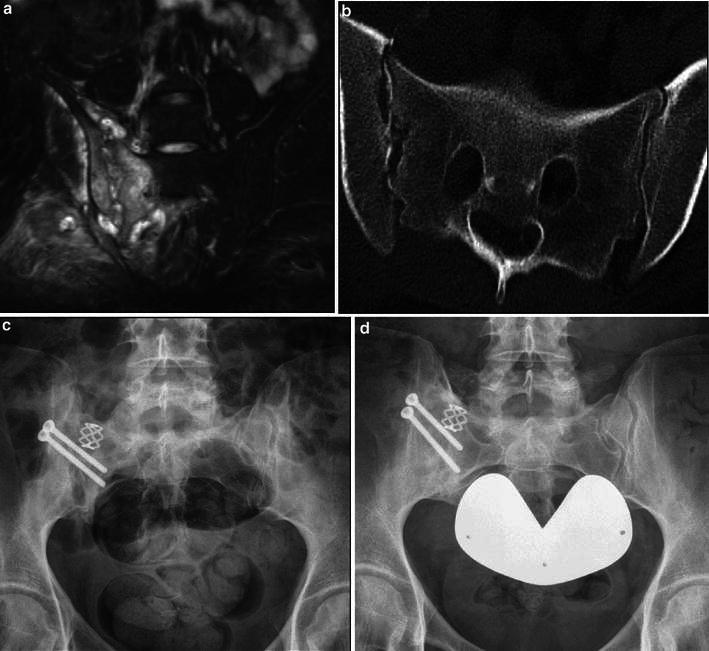
**a** Case 9: MRI performed after admission showed high signal intensity in the right SIJ and adjacent muscles with abscess formation and bone oedema. **b** CT revealed widening of the joint space, cavitations and sequestrum formation. **c** Postoperative radiograph revealed good position of the cage and screws. The patient was allowed to bear weight with assistance after 6 weeks and to fully bear weight after 4 months, after confirmation of bony fusion of the joint. After 1 year, the patient had no complaints and was satisfied. **d** FU radiographs showed complete bony fusion of the joint. At the last FU visit (49 months postoperatively), she had excellent functional outcome, no pain and no limitations of daily activity. She returned to work and practised sport regularly

**Fig. 3:**
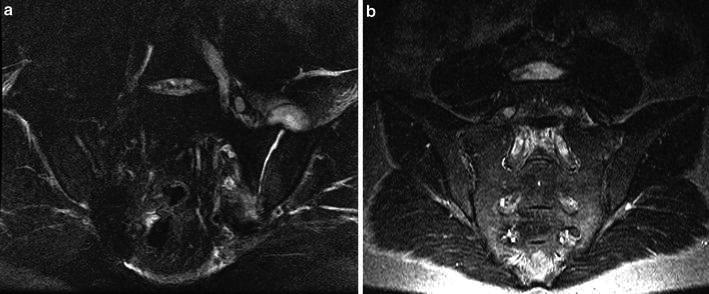
**a** Case 12: MRI performed 1 week after onset of the patient’s symptoms showed high signal intensity in the left SIJ and iliacus muscle with abscess formation. The patient was operated by combined anterior and posterior debridement. Full mobilisation was allowed after 2 weeks. The patient was satisfied. **b** FU MRI after 2 months revealed no more abnormal inflammatory signals. At the last FU visit after 80 months, the patient had excellent functional outcome

**Fig. 4:**
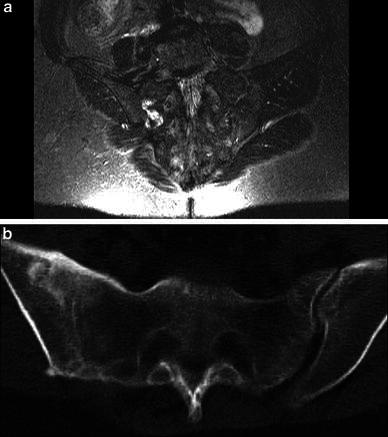
**a** Case 11: Preoperative MRI showed localised area of high signal inflammatory intensity in the right SIJ. The SIJ was debrided posteriorly. The patient was allowed to fully bear weight after 2 weeks. **b** CT confirmed solid joint fusion after 1 year. The last clinical FU after 86 months showed excellent outcome, no pain and normal daily activities

Our clinical results were excellent or good in 13 patients (65 %), these results being comparable to those of Schubert et al. [17], who performed debridement and primary arthrodesis in nine patients with pyogenic SIJ infections (Figs. 2, 3, 4).

**Fig. 5:**
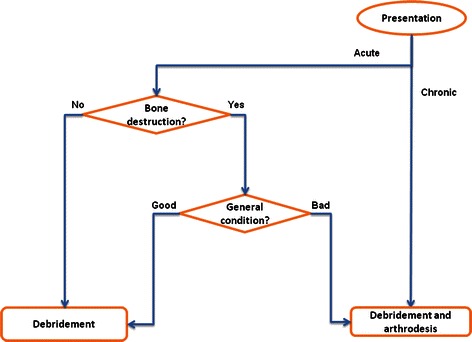
Flowchart of the recommended treatment pathway

There is debate over whether to perform arthrodesis of the joint or to limit surgery to drainage of the abscess and debridement of the joint. The operative management of SIJ infections, from our experience, consists of debridement in cases of acute soft tissue infection or cases of mild bone destruction. Joint arthrodesis is recommended in generally ill patients even with mild joint destruction for early assisted mobilisation as well as in patients with chronic joint affection (Fig. 5).

In acute cases, the primary aim should be to save joint integrity by early debridement, depending on joint destruction and general patient condition. When it is chronic, it is not secure only to debride the joint, which should be fused.
